# Bone tissue, blood lipids and inflammatory profiles in adolescent male athletes from sports contrasting in mechanical load

**DOI:** 10.1371/journal.pone.0180357

**Published:** 2017-06-29

**Authors:** Ricardo R. Agostinete, João P. Duarte, João Valente-dos-Santos, Manuel J. Coelho-e-Silva, Oscar M. Tavares, Jorge M. Conde, Carlos A. Fontes-Ribeiro, Giancarlo Condello, Laura Capranica, Suziane U. Caires, Rômulo A. Fernandes

**Affiliations:** 1 Department of Physical Education, Laboratory of Investigation in Exercise (LIVE), São Paulo State University (UNESP), São Paulo, Brazil; 2 Department of Physical Therapy, Post-Graduation Program in Physical Therapy, São Paulo State University (UNESP), São Paulo, Brazil; 3 CIDAF (UID/DTP/04213/2016), Faculty of Sport Sciences and Physical Education, University of Coimbra, Coimbra, Portugal; 4 Portuguese Foundation for Science and Technology (SFRH/BD/101083/2014), Lisbon, Portugal; 5 Portuguese Foundation for Science and Technology (SFRH/BPD/100470/2014), Lisbon, Portugal; 6 Institute for Biomedical Imaging and Life Sciences (IBILI), Faculty of Medicine, University of Coimbra, Coimbra, Portugal; 7 Faculty of Physical Education and Sport, Lusófona University of Humanities and Technologies, Lisbon, Portugal; 8 Department of Medical Imaging and Radiation Therapy, School of Health and Technology, Instituto Politécnico de Coimbra, Coimbra, Portugal; 9 Instituto Politécnico de Coimbra, Coimbra, Portugal; 10 Institute of Pharmacology and Experimental Therapeutics, Faculty of Medicine, University of Coimbra, Coimbra, Portugal; 11 Department of Movement, Human and Health Sciences, University of Rome Foro Italico, Rome, Italy; 12 Post-Graduation Program in Kinesiology, Institute of Biosciences, São Paulo State University (UNESP), São Paulo, Brazil; University of Oulu, FINLAND

## Abstract

Exploring the effect of non-impact and impact sports is particular relevant to understand the interaction between skeletal muscle and bone health during growth. The current study aimed to compare total and regional bone and soft-tissue composition, in parallel to measurements of blood lipid and inflammatory profiles between adolescent athletes and non-athletes. Anthropometry, biological maturity, dual energy X-ray absorptiometry (DXA) scans, training load and lipid and inflammatory profiles were assessed in a cross-sectional sample of 53 male adolescents (20 non-athletes, 15 swimmers and 18 basketball players) aged 12–19 years. Multiple comparisons between groups were performed using analysis of variance, covariance and magnitude effects (*ES-r* and Cohen’s *d*). The comparisons of controls with other groups were very large for high-sensitivity C-reactive protein (*d* range: 2.17–2.92). The differences between sports disciplines, regarding tissue outputs obtained from DXA scan were moderate for all variables except fat tissue (*d* = 0.4). It was possible to determine small differences (*ES-r* = 0.17) between controls and swimmers for bone area at the lower limbs (13.0%). In parallel, between swimmers and basketball players, the gradient of the differences was small (*ES-r* range: 0.15–0.23) for bone mineral content (24.6%), bone area (11.3%) and bone mineral density (11.1%) at the lower limbs, favoring the basketball players. These observations highlight that youth male athletes presented better blood and soft tissues profiles with respect to controls. Furthermore, sport-specific differences emerged for the lower limbs, with basketball players presenting higher bone mineral content, area and density than swimmers.

## Introduction

Regular participation in sport is a feature of the daily lives of youth. Evidence based on three day diary records in boys and girls 12–14 years [1], accelerometry in boys 6–12 years [2], questionnaires in U.S. boys and girls 11–12 years [3] and girls 13–18 years [4], and questionnaires in Finnish twins 16–18 years [5] indicate higher levels of physical activity in sport participants compared to non-participants. In fact, regular engagement in physical exercise (e.g., 60 minutes of moderate- to vigorous-intensity physical activity daily) is recommended for youth to develop healthy musculoskeletal tissues, cardiovascular system, and to maintain healthy blood parameters and body weight [6]. A recent study compared the effects of a 6-month soccer programme and a traditional physical activity programme on changes in body composition, cardiometabolic, inflammatory and oxidative stress markers, in parallel to measurements of cardiorespiratory fitness in obese boys [7]. After the intervention, both groups had significantly lower relative fatness and waist circumference, total cholesterol, and improved cardiorespiratory fitness. The results also suggested that soccer-based intervention was more effective for the reduction of childhood obesity and associated consequences. Given the seemingly central role of competitive sport in the developmental process of youth, a question of relevance is the sport-related long-term benefits of participation.

Adolescence is considered a crucial period for bone formation and the literature suggested that about 90% of bone mass observed in adulthood is acquired during early life stages [8]. By inference, it is expected that children and adolescents with low levels of bone mineral have an increased likelihood of developing osteoporosis [9], which is a chronic disease with a great socio-economic impact [10] and currently considered as the worldwide second cause of disability affecting one third of women and one out of eight men 50 years [11]. Acquisition of bone mineral content during the first two decades of life should be considered a relevant public health strategy and exercise is considered an effective determinant to increase the peak bone mass during early life [12], in addition to genetic, hormonal and nutritional determinants.

Existing recommendations for physical activity focus on the manipulation of frequency, intensity and volume aiming to improve bone health. The tensile and compressive forces associated with muscular contractions during weight-bearing activities and specialized exercises such as strength and resistance training consistently have a favourable influence on skeletal tissue [13–15] and comparisons of elite young athletes with less active youth [16, 17] indicate a beneficial effect of physical activity on skeletal health. The osteogenic influence of physical activity is generally sites specific and related to local mechanical strains. The benefits are reflected in bone mineral content, bone mineral density, and apparent bone mineral density. A systematic review conducted by Strong et al. [18] concluded that the benefits are not as consistently established for adolescents in later stages of puberty. The literature suggests improvement in health outcomes in association with physical activity in obese and non-obese youth but the dose of physical activity that is necessary to prevent or treat may be specific to each health outcome [18].

In general, recommendations support physical activity to maintain healthy body weight and lipid profiles [19, 20] relevant to prevent from childhood obesity and risks of coronary heart diseases and atherosclerosis [21]. However, distinct sport-based programs could lead to different responses. According to Strong et al. [18], youth engaging in ≥4-month of regular physical exercise encompassing at least 40-min of moderate- to vigorous-intensity activities could have beneficial effects on triglyceride and high density lipoprotein cholesterol (HDL-c) levels, whereas effects on total cholesterol and low density lipoprotein cholesterol (LDL-c) levels resulted not consistent. Furthermore, high intensity exercise could promote inflammatory cytokines releases, such as the C-reactive protein (CRP), which negatively impacts the somatic growth of children and adolescents [22].

The present study was aimed to compare whole-body and regional indicators of bone tissue, in parallel to measurements of blood lipid and inflammatory profiles in adolescent athletes contrasting in mechanical load (basketball and swimming athletes) and non-athlete counterparts. It is hypothesized that sport-associated variation are specific to health outcomes and when regarding measurements of bone mineral content, differences between athletes and non-athletes are generalized while between swimmers and basketball players groups are more apparent in specific regions (upper arms, trunk, lower limbs).

## Materials and methods

### Ethics statement

All procedures were approved by the ethical board of the Sao Paulo State University (CAAE, 02891112.6.0000.5402) and were conducted in accordance with the Declaration of Helsinki for human studies by the World Medical Association. After approval obtained from the Municipal Department of Education and Department of Sports to collect data in schools and sport clubs, respectively, parents or legal guardians completed a written statement of consent. Participants were informed about the objectives and methodology and also that participation was voluntary and that they could withdraw from the experiment at any time.

### Participants

The final sample was composed of 53 male adolescents (S1 File). Twenty adolescents composed the Control Group (recruited in several public and private schools) while 33 adolescents were athletes (Basketball, n = 18; Swimming, n = 15). Swimmers and basketball players were recruited in two clubs registered in the respective national federations.

The inclusion criteria for this study were: (*i*) chronological age between 10 and 19 years; (*ii*) male sex; (*iii*) no use of medication that could affect bone metabolism; (*iv*) absence of previous or current bone fractures. Furthermore, to be included in the control group, participants had to declare to participate in regular physical education classes (two hours for^.^week^-1^) with no previous or actual engagement in organized sports.

### Anthropometry

Stature and sitting height were recorded with a stadiometer (Sanny, model American Medical of the Brazil Ltda, Brazil) and a table (Harpenden sitting height table, model 98.607, Holtain Ltd, Crosswell, UK) to the nearest 0.1 cm. Leg length was estimated as stature minus sitting height. Body mass was measured using a digital scale (Filizzola PL 150, model Filizzola Ltda, Brazil) to the nearest 0.1 kg. A trained technician performed all measurements during a single visit to the laboratory. Finally, sitting height and body mass was used to calculate the individual body mass index (BMI, m^2^/kg).

### Biological maturity

The maturity offset was calculated [23]. It corresponds to an algorithm derived from two longitudinal studies of Canadian youth and one of Belgian twins was used to predict the time (years) from/to age peak of height velocity (APHV), labeled maturity offset. Then, predicted APHV (years) was estimated as chronological age minus maturity offset.

$$\mathrm{Maturity}\;\mathrm{offset}\;(\mathrm{in}\;\mathrm{years})=-9.236+(0.0002708\times (\mathrm{Leg}\;\mathrm{length}\times \mathrm{Sitting}\;\mathrm{height}))+(-0.001663\times (\mathrm{Age}\times \mathrm{Leg}\;\mathrm{length}))+(0.007216\times (\mathrm{Age}\times \mathrm{Sitting}\;\mathrm{height}))+(0.02292\times \left(\frac{\mathrm{Body mass}}{\mathrm{Stature}}\times 100\right))$$ (1)

### Dual X-ray energy absorptiometry (DXA)

Total tissue (kg), fat tissue (kg), lean soft tissue (kg), bone mineral content (BMC, g), bone mineral density (BMD, g/cm^2^) and bone area (in cm^2^) were assessed using DXA (Lunar DPX-NT; General Electric Healthcare, Little Chalfont, Buckinghamshire, UK) and data for head, upper limbs, lower limbs, trunk (ribs, pelvis and column) and whole body were processed by means of a GE Medical System Lunar software (version 4.7). A certified technician calibrated the equipment on a daily basis and performed the scans with the participant wearing light clothes (short and t-shirt) and laying in the supine position on the apparatus (approximately 15 minutes). The precision of the machine in terms of coefficient of variation was 0.66% (n = 30 subjects not involved in this study).

### Training routine

The athletes reported their experience in the sports (in years) and the coaches reported the minutes trained per day and sessions trained per week by each athlete (weekly training volume). None of the athletes involved in the study performed another supervised physical exercise.

### Training load

Thirty minutes after the end of the training, according literature recommendation [24], athletes were administered the CR-10 rating of perceived exertion (RPE) scale [25] adapted by Foster et al.[26] and reported the training duration (min). Thus, training load per day was calculated multiplying the score of the RPE with the total duration of the training section (in minutes)[24, 26] and summated to obtain the monthly training load.

### Vitamin D intake

The adolescents reported through of a questionnaire (listed with foods rich in vitamin D usually present in a Brazilian diet) the frequency of consumption of foods rich (Likert scale) in vitamin D in a week, considering the previous week of the evaluation. The sums of the values of scale were considered as a vitamin D intake score.

### Lipid and inflammatory profiles

Blood samples were collected during a rest day following a training session. A certified technician collected blood samples in the morning after a 12-hour fasting. An autohumalyzer (Dimension RxL Max; Siemens Dade-Behring, Deerfield, Illinois) and an enzymatic colorimetric kit were used to assess total cholesterol, HDL-c, LDL-c, very-low density lipoprotein cholesterol (VLDL-c) and triacylglycerol. In addition, high-sensitivity (H-S) CRP was assessed through the turbidimetric method (LabMax 240; Chema Diagnostica, Monsano, Italy) using an enzyme kit (Millipore, St. Charles, Missouri) with intra- and inter-assay reliability ranging between 4.6 and 6.0%, respectively.

### Statistical analysis

For the descriptive statistic was composed of mean, standard deviation, median, range (minimum and maximum value), mean (and respective standard error of the mean plus 95% confidence interval) and standard deviation. Normal distribution was examined using the test Kolmogorov-Smirnov. Analysis of variance (ANOVA) was used to assess the effect of sport participation on anthropometry, maturation, metabolic outputs, obtained from blood samples, and variables derived from the DXA assessments. Additionally, taking into account bone parameters as outcome, multivariate models adjusted by chronological age, maturity offset, score of vitamin D and weekly training volume have been created using analysis of covariance (ANCOVA). The associated magnitude effect among independent and dependent variables were determined by means of the effect size correlations (ES-*r*), which is estimated using the square root of the ratio of the F-value squared and the difference between the F-value squared and degrees of freedom. Coefficients were interpreted as follows: trivial (r > < 0.1), small (0.1 < r < 0.3), moderate (0.3 < r < 0.5), large (0.5 < r < 0.7), very large (0.7 > < r > < 0.9), nearly perfect (r > < 0.9) and perfect (r = 1) [27]. Additionally, multiple comparisons between groups (controls vs. swimmers; controls vs. basketball players, swimmers vs. basketball players) were performed by calculating Cohen’s *d* effect sizes [28]. Thresholds were interpreted as follows: 0.2, 0.6, 1.2, 2.0, 4.0 for small, moderate, large, very large and extremely large differences, respectively [27]. Statistical significance was set at p-value <0.05 and all analyses were performed using the statistical software SPSS (version 24.0).

## Results

The descriptive characteristics and results of the Kolmogorov-Smirnov test for age, biological maturity, training experience, indicators of lipid and inflammatory profile, anthropometry of the overall body size and outputs of the whole body DXA assessments, for the total sample, are presented in Table 1. Descriptive statistics for DXA regional body composition are summarized in Table 2.

**Table 1:** Descriptive statistics on chronological age, maturation, training experience, parameters of training experience, indicators of lipid profile plus inflammatory biomarker, anthropometry of the overall body size and outputs of whole body DXA assessments for the total sample (n = 53).

Variable	unit	Descriptive							Normality (kolmogorov-Smirnov)	
		Range		Mean				Standard deviation		
		minimum	maximum	value	SEM	95% CI				
						lower	upper		K-S value	p
Chronological age	years	12	19	14.34	0.26	13.83	14.85	1.92	0.191	<0.001
Maturity offset	years	-2.6	2.9	0.08	0.21	-0.34	0.51	1.55	0.100	0.200
Age at peak height velocity	years	12.7	16.8	14.25	0.11	14.03	14.47	0.81	0.094	0.200
Vitamin D	score	0	14	6.4	0.49	5.40	7.39	3.6	0.161	0.002
Sport experience ^a^	years	2.0	13.4	5.66	0.5	4.60	6.73	3.00	0.136	0.126
Training sessions per week ^a^	sessions	2	6	5.55	0.169	5.20	5.89	0.971	0.438	<0.001
Training time per week ^a^	min	720	2160	1108	47.8	1010	1205	274	0.290	<0.001
Training load during past month ^a^		2880	27930	13863	1037	11747	15979	5868	0.174	0.015
Body mass	kg	41.6	91.1	67.6	1.59	64.3	70.8	11.6	0.063	0.200
Stature	cm	150.4	199.7	174.2	1.64	170.9	177.5	11.9	0.073	0.200
Sitting height	cm	76.0	101.3	89.2	0.87	87.5	91.0	6.3	0.079	0.200
Estimated leg length	cm	70.2	104.9	84.9	0.95	83.0	86.8	6.9	0.079	0.200
Total cholesterol	mg/dL	103	208	147.8	3.0	141.6	154.0	22.5	0.093	0.200
HDL-c	mg/dL	32	73	46.5	1.1	44.31	48.82	8.1	0.109	0.169
LDL-c	mg/dL	49.4	137.2	88.4	2.5	83.3	93.6	18.5	0.092	0.200
VLDL-c	mg/dL	4.7	49.5	12.7	1.1	10.5	15.0	8.0	0.235	<0.001
Triglycerides	mg/dL	23.7	247.4	63.9	5.5	52.8	75.0	40.2	0.235	<0.001
HS C-reactive protein	mg/L	0.24	14.78	3.42	0.3	2.7	4.0	2.3	0.176	<0.001
DXA, whole-body										
Total tissue	kg	40.6	91.0	66.8	1.61	63.6	70.0	11.7	0.069	0.200
Fat tissue	kg	3.2	33.1	13.2	1.10	11.0	15.4	8.0	0.152	0.004
Lean soft tissue	kg	27.2	74.2	50.7	1.63	47.4	53.9	11.9	0.108	0.178
Bone mineral content	g	1634	4800	2883	101.1	2680	3086	736	0.106	0.200
Bone area	Cm^2^	1676	3078	2411	46	2319	2504	336	0.081	0.200
Bone mineral density	g/cm^2^	0.921	1.559	1.185	0.020	1.144	1.225	0.146	0.093	0.200

Abbreviations: SEM, standard error of the mean; 95%CI, 95% confidence intervals; HDL-c, high-density lipoprotein cholesterol; LDL-c, low-density lipoprotein cholesterol; VLDL-c, very-low-density lipoprotein cholesterol; HS C-reactive protein, high-sensitivity C-reactive protein; DXA, dual-energy X-ray absorptiometry.

^a^ Uniquely for swimmers and basketball players.

**Table 2:** Descriptive statistics for variables reporting inter-individual variability on DXA assessments on trunk, upper limbs and lower limbs (n = 53).

Variable	unit	Descriptive							Normality (kolmogorov-Smirnov)	
		Range		Mean				Standard deviation		
		minimum	maximum	value	SEM	95% CI				
						lower	upper		K-S value	p
***Total tissue***										
Trunk	kg	16.8	39.9	29.9	0.7	28.4	31.3	5.4	0.083	0.200
Upper limbs	kg	3.5	10.3	7.2	0.2	6.7	7.6	1.7	0.114	0.085
Lower limbs	kg	15.7	34.9	24.9	0.6	23.6	26.2	4.7	0.067	0.200
***Fat tissue***										
Trunk	kg	1.6	16.9	6.6	0.5	5.6	7.7	3.8	0.139	0.012
Upper limbs	kg	0.1	2.5	0.7	0.08	0.5	0.9	0.6	0.216	0.000
Lower limbs	kg	0.9	13.6	5.2	0.4	4.3	6.2	3.4	0.154	0.003
***Lean soft tissue***										
Trunk	kg	12.1	31.9	22.2	0.7	20.7	23.7	5.4	0.126	0.036
Upper limbs	kg	2.6	9.1	6.0	0.2	5.5	6.5	1.8	0.097	0.200
Lower limbs	kg	9.4	28.2	18.4	0.6	17.2	19.7	4.4	0.079	0.200
***Bone mineral content***										
Trunk	g	423	1531	907	36	834	980	265	0.089	0.200
Upper limbs	g	168	603	373	16	340	407	121	0.122	0.046
Lower limbs	g	641	2104	1175	42	1090	1261	310	0.081	0.200
***Bone area***										
Trunk	cm^2^	576	1191	890.7	21	848	933	154	0.085	0.200
Upper limbs	cm^2^	263	560	415	10	393	437	78	0.089	0.200
Lower limbs	cm^2^	581	1153	865	16	832	897	117	0.062	0.200
***Bone mineral density***										
Trunk	g/cm^2^	0.730	1.286	0.998	0.018	0.961	1.034	0.132	0.094	0.200
Upper limbs	g/cm^2^	0.638	1.281	0.878	0.022	0.833	0.923	0.162	0.113	0.090
Lower limbs	g/cm^2^	1.011	1.981	1.339	0.027	1.284	1.394	0.200	0.078	0.200

Abbreviations: SEM, standard error of the mean; 95%CL, 95% confidence intervals.

Group-related variation for chronological age, maturation, training experience, indicators of lipid profile plus inflammatory biomarker, anthropometry of the overall body size and outputs of whole body DXA assessments is present in Table 3. The basketball players reported an accumulated training experience of 4.3±2.3 (95%CI = 3.1–5.4) years, an actual training volume of 5.3±1.1 (95%CI = 4.7–5.9) days^.^week^-1^ and a cumulated weekly training of 1072±123 (95%CI = 1010–1133) min. The swimmers reported an accumulated training experience of 7.2±2.9 (95%CI = 5.6–8.9) years, an actual training volume of 5.8±0.5 (95%CI = 5.4–6.1) days^.^week^-1^, and a cumulated weekly training of 1152±387 (95%CI = 937–1366) min.

**Table 3:** Means and standard deviations by group and results of ANOVA to test the effect of sport participation on chronological age, maturation, training experience, indicators of lipid profile plus inflammatory biomarker, anthropometry of the overall body size and outputs of whole body DXA assessments.

Dependent variable		X: independent variable (Sport participation)			ANOVA			
Y_i_:	unit	Controls (n = 20)	Swimmers (n = 15)	Basketball players (n = 18)	F	p	Magnitude effect	
							ES-r	(qualitative)
Chronological age	years	13.0 ± 1.3	15.9 ± 2.1 ^b^^,^^c^	14.5 ± 0.9 ^b^^,^^d^	15.850	<0.001	0.388	(moderate)
Maturity offset	years	-1.2 ± 1.0	1.1 ± 1.5 ^b^	0.7 ± 0.74 ^b^	23.939	<0.001	0.489	(moderate)
Age at peak height velocity	years	14.2± 0.4	14.8 ± 0.9 ^c^	13.7 ± 0.6 ^d^	9.733	<0.001	0.280	(small)
Vitamin D	score	9.5 ± 3.3	4.3 ± 2.0 ^b^	4.6 ± 2.4 ^b^	20.838	<0.001	0.455	(moderate)
Training experience ^a^	years	-	7.2 ± 2.9 ^c^	4.3 ± 2.3 ^d^	-	-	-	-
Training per week ^a^	sessions	-	5.8 ± 0.5	5.3 ± 1.1	-	-	-	-
Training per week ^a^	min	-	1152 ± 387.6	1072 ± 122.9	-	-	-	-
Training load past month ^a^		-	15991 ± 6577	11986 ± 4569	-	-	-	-
Log-transformed TLPM ^a^		-	4.16 ± 0.2	4.04 ± 0.2	-	-	-	-
Body mass	kg	61.2 ± 13.0	66.2 ± 12.7 ^c^	75.7 ± 9.8 ^b^^,^^d^	10.130	<0.001	0.288	(small)
Stature	cm	164.0 ± 7.5	174.9 ± 8.5 ^b^^,^^c^	185.0 ± 8.2 ^b^^,^^d^	32.045	<0.001	0.562	(large)
Sitting height	cm	83.5 ± 4.2	91 ± 5.0 ^b^	94.1 ± 3.9 ^b^	29.520	<0.001	0.541	(large)
Estimated leg length	cm	80.4 ± 5.6	83.9 ± 4.2 ^c^	90.8 ± 6.0 ^b^^,^^d^	17.506	<0.001	0.412	(moderate)
Total cholesterol	mg/dL	153.7 ± 20.9	151.4 ± 17.9	138.3 ± 25.5	2.636	0.082	0.095	(small)
HDL	mg/dL	44.3 ± 5.0	50.9± 9.3 ^b^	45.3 ± 8.9	3.416	0.041	0.120	(small)
LDL	mg/dL	91.5 ± 19.4	90.8 ± 14.1	83.0 ± 20.4	1.168	0.319	0.045	(small)
VLDL	mg/dL	17.7 ± 10.8	9.5 ± 2.3 ^b^	9.8 ± 3.9 ^b^	7.848	0.001	0.239	(small)
Triglycerides	mg/dL	88.9 ± 54.0	47.8 ± 11.9 ^b^	49.4 ± 19.5 ^b^	7.860	0.001	0.239	(small)
HS C-reactive protein	mg/L	1.3 ± 1.0	3.9± 0.6 ^b^^,^^c^	5.3 ± 2.5 ^b^^,^^d^	28.464	<0.001	0.537	(large)
DXA, whole-body								
Total tissue	kg	60.1 ± 8.1	65.7 ± 12.1	75.2 ± 9.8 ^b^^,^^d^	10.926	<0.001	0.304	(moderate)
Fat tissue	kg	18.6 ± 8.8	8.7 ± 4.9 ^b^	11.0 ± 5.6 ^b^	10.260	<0.001	0.291	(small)
Lean soft tissue	kg	39.1 ± 6.9	54.0 ± 8.2 ^b^^,^^c^	60.6 ± 7.2 ^b^^,^^d^	41.706	<0.001	0.625	(large)
Bone mineral content *	g	2941 ± 194	2664 ± 143	3001 ± 142	2.863	0.067	0.111	(small)
Bone area *	cm^2^	2370 ± 194	2324 ± 143	2419± 141	0.221	0.803	0.010	(trivial)
Bone mineral density *	g/cm^2^	1.198 ± 0.048	1.134± 0.035	1.213 ± 0.035	2.621	0.084	0.102	(small)

Abbreviations: HDL-c, high-density lipoprotein cholesterol; LDL-c, low-density lipoprotein cholesterol; VLDL-c, very-low-density lipoprotein cholesterol; HS C-reactive protein, high-sensitivity C-reactive protein; DXA, dual-energy X-ray absorptiometry; *ES-r*, effect size correlation.

* ANCOVA model adjusted by chronological age, maturity offset, score of vitamin D intake and weekly training volume (data presented as mean ± SEM).

^a^ Compare means for two groups using independent t-test.

^b^ p-value <0.05 compared to controls.

^c^ p-value <0.05 compared to basketball players

^d^ p-value <0.05 compared to swimmers.

**Fig 1:**
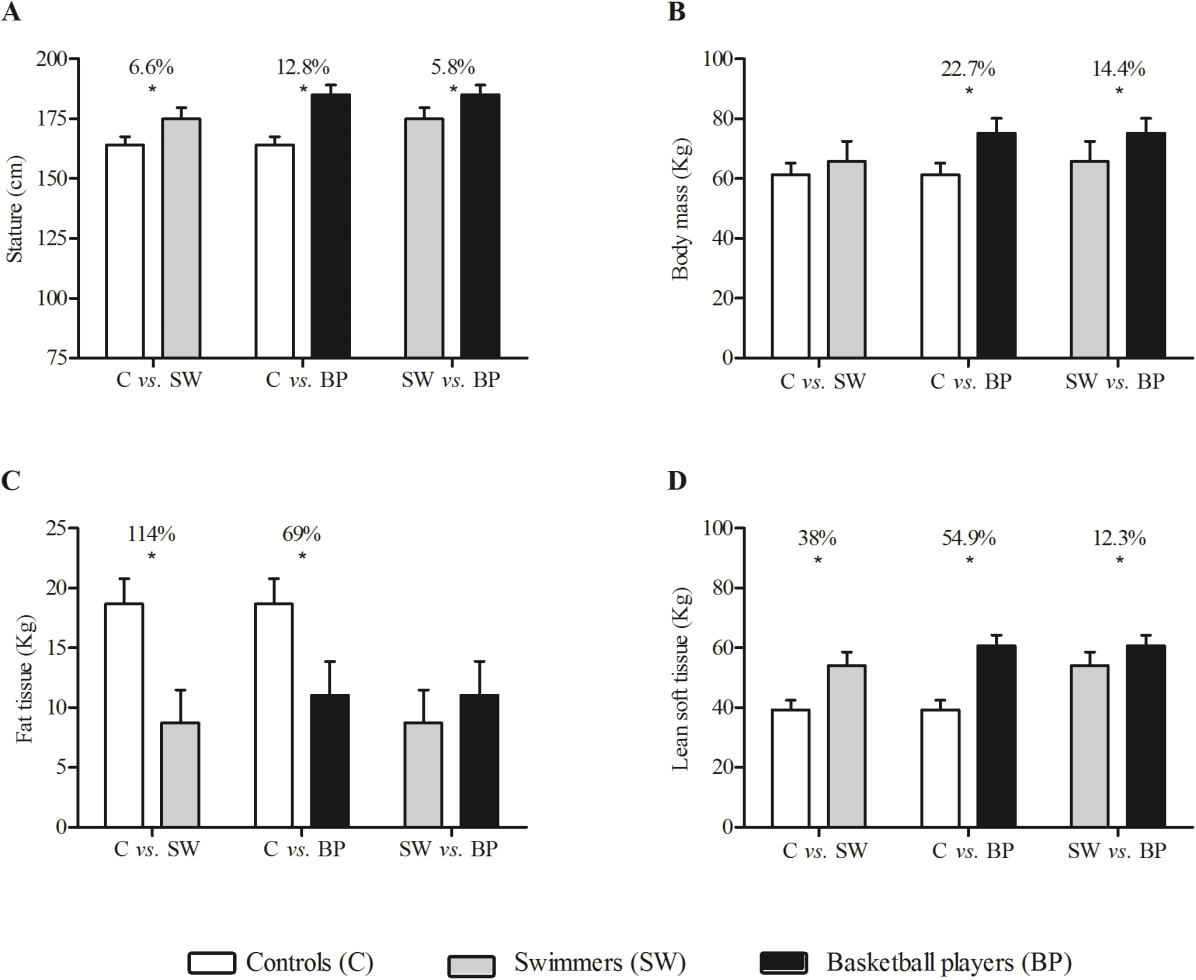
**Stature (panel A), body mass (panel B), fat tissue (panel C) and lean soft tissue (panel D) in male controls (white bars), swimmers (grey bars) and basketball players (black bars).** * indicates difference between the groups (p<0.05).

Controls were younger (13.0±1.3, 95%CI = 12.3–13.6 years) and showed a lower maturity offset (-1.2±1.0, 95%CI = -1.7–-0.7 yrs), compared to basketball players (age: 14.5±0.9, 95%CI = 14.0–14.9 years; maturity offset: 0.7±0.7, 95%CI = 0.3–1.1 years) and swimmers (age: 15.9±2.1, 95%CI = 14.7–17.1 years; maturity offset: 1.1±1.5, 95%CI = 0.2–1.9 years) counterparts (p<0.001, ES-r range: 0.4–05). Furthermore, APHV showed a difference (p<0.001, ES-r = 0.3) only between swimmers (14.8±0.9, 95%CI = 14.2–15.3 years) and basketball players (13.7±0.6, 95%CI = 13.4–14.0 years), with controls showing intermediate values (14.2±0.4, 95%CI = 14.0–14.5 yrs). In general, basketball players showed highest stature, body mass, and lean soft tissue values, swimmers intermediate values, and controls the lowest values. Fig 1 shows the relative differences (p<0.05) between groups for stature (range: 5.8–12.8%), body mass (range: 14.4–22.7%), fat tissue (range: 69–114%) and lean soft tissue (range: 12.3–54.9%). Finally, 45% of controls were categorized as normal weight, 45% overweight, and 10% as obese. Conversely, athletes of swimmers were categorized as 86.7% normal weight and 13.3% as overweight. The basketball players were categorized 61.1% as normal weight and 38.9% as overweight. The whole body bone variables were similar among the groups.

Concerning the blood parameters, only total cholesterol and LDL-c showed no difference between groups (Table 3). Conversely, controls showed differences with respect to the athletes for triacylglycerol (p = 0.001, ES-r = 0.2) and HS-CRP (p<0.001, ES-r = 0.5), and for HDL (p = 0.04, ES-r = 0.1) with respect to swimmers only.

The effect of sport participation on regional soft tissue and bone variables is presented in Table 4. Soft tissue differences emerged mainly between the athletes and controls (p<0.01; *ES-r* range: 0.2–0.6). However, for bone variables differences were manly noted between the sports participation groups at the lower limbs (p<0.05; *ES-r* range: 0.1–0.2).

**Table 4:** Means and standard deviations by group and results of ANOVA to test the effect of sport participation on DXA assessments on trunk, upper limbs and lower limbs.

Dependent variable		X: independent variable (Sport participation)			ANOVA			
Y_i_:	unit	Controls (n = 20)	Swimmers (n = 15)	Basketball players (n = 18)	F	p	magnitude effect	
							*ES-r*	(qualitative)
***Total tissue***								
Trunk	kg	26.5 ± 3.8	30.4 ± 6.1 ^a^	33.1 ± 4.1 ^a^	9.742	<0.001	0.280	(small)
Upper limbs	kg	5.7 ± 1.0	7.7 ± 1.6 ^a^	8.3 ± 1.2 ^a^	21.712	<0.001	0.465	(moderate)
Lower limbs	kg	23.2 ± 3.4	22.8 ± 4.3	28.5 ± 4.3 ^a^^,^^b^	10.976	<0.001	0.305	(moderate)
***Fat tissue***								
Trunk	kg	8.9 ± 4.4	4.8 ± 2.9 ^a^	5.7 ± 2.8 ^a^	6.845	0.002	0.215	(small)
Upper limbs	kg	1.2± 0.7	0.4 ± 0.3 ^a^	0.5 ± 0.3 ^a^	12.394	<0.001	0.331	(moderate)
Lower limbs	kg	7.7 ± 3.7	3.0 ± 1.7 ^a^	4.4 ± 2.4 ^a^	13.226	<0.001	0.346	(moderate)
***Lean soft tissue***								
Trunk	kg	16.8 ± 3.0	24.6 ± 3.8 ^a^	26.3 ± 3.2 ^a^	42.354	<0.001	0.629	(large)
Upper limbs	kg	4.2 ± 1.1	6.8 ± 1.3 ^a^	7.4 ± 1.1 ^a^	39.392	<0.001	0.612	(large)
Lower limbs	kg	14.5 ± 2.6	18.7 ± 2.9 ^a^	22.6± 2.9 ^a^^,^^b^	39.205	<0.001	0.611	(large)
***Bone mineral content*** *								
Trunk	g	952 ± 78	837 ± 57	915 ± 56	1.138	0.329	0.047	(trivial)
Upper limbs	g	359 ± 31	375 ± 23	387 ± 23	0.218	0.805	0.009	(trivial)
Lower limbs	g	1238 ± 92	1002 ± 68	1250 ± 67 ^b^	6.957	0.002	0.232	(small)
***Bone area*** *								
Trunk	cm^2^	904 ± 46	874 ± 34	888 ± 34	0.143	0.867	0.006	(trivial)
Upper limbs	cm^2^	390 ± 26	429 ± 19	430 ± 19	0.448	0.642	0.019	(trivial)
Lower limbs	cm^2^	899 ± 42	795 ± 31 ^a^	885 ± 30 ^b^	4.681	0.014	0.169	(small)
Bone mineral density*								
Trunk	g/cm^2^	1.019 ±0.042	0.944 ± 0.031	1.020 ± 0.031	3.179	0.051	0.121	(small)
Upper limbs	g/cm^2^	0.875 ± 0.051	0.862 ± 0.037	0.895 ± 0.037	0.413	0.664	0.018	(trivial)
Lower limbs	g/cm^2^	1.350 ± 0.068	1.257 ± 0.050	1.397 ± 0.049 ^b^	4.049	0.024	0.150	(small)

Abbreviations: *ES-r*, effect size correlation.

* ANCOVA model adjusted by chronological age, maturity offset, score of vitamin D intake and weekly training volume (data presented as mean ± SEM).

^a^ p-value <0.05 compared to controls.

^b^ p-value <0.05 compared to swimmers.

**Fig 2:**
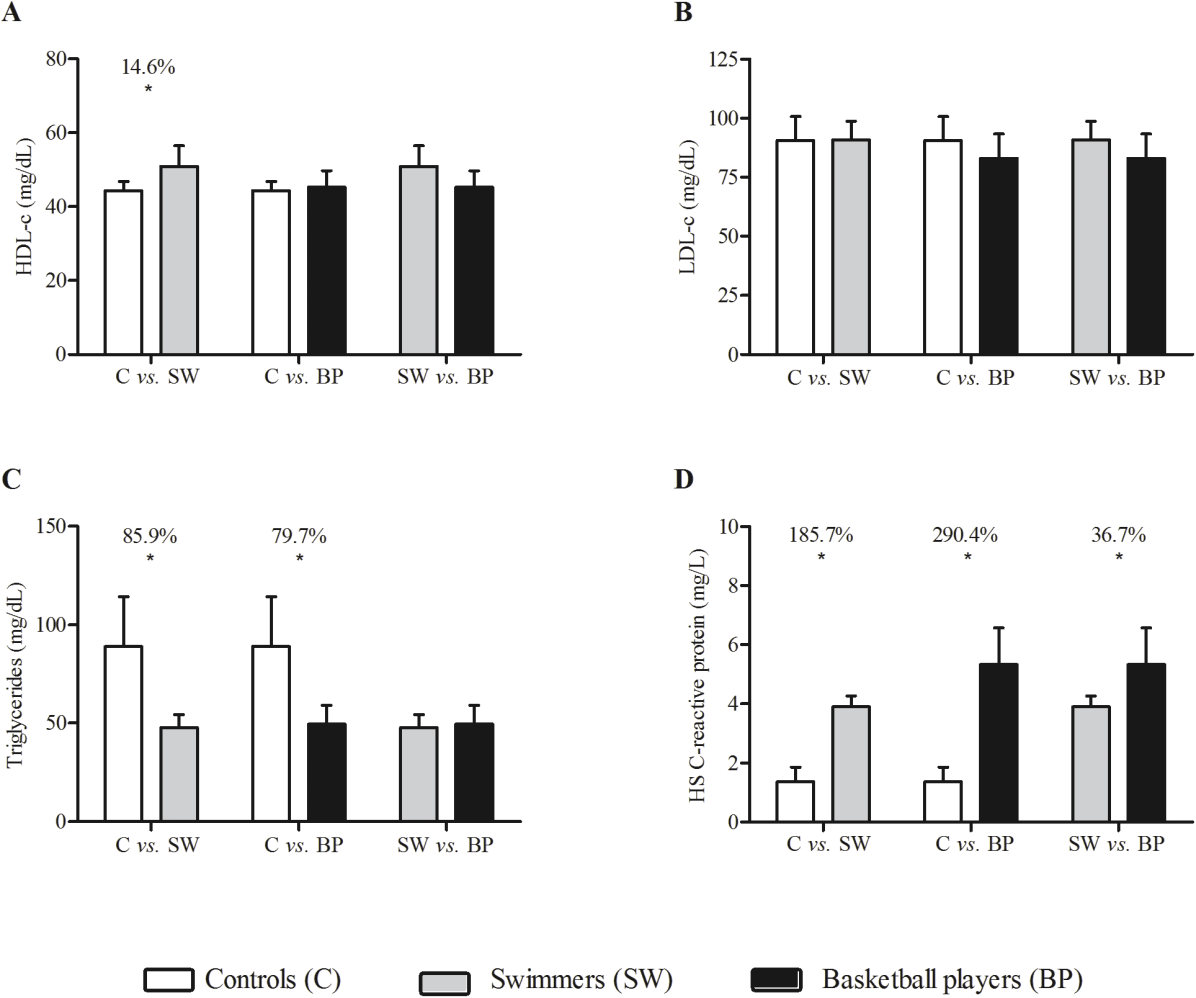
High-density lipoprotein-cholesterol (HDL-c), low-density lipoprotein cholesterol (LDL-c), triglycerides and high-sensitivity C-reactive protein (hsCRP) in male controls (white bars), swimmers (grey bars) and basketball players (black bars). * indicates difference between the groups (p<0.05).

**Fig 3:**
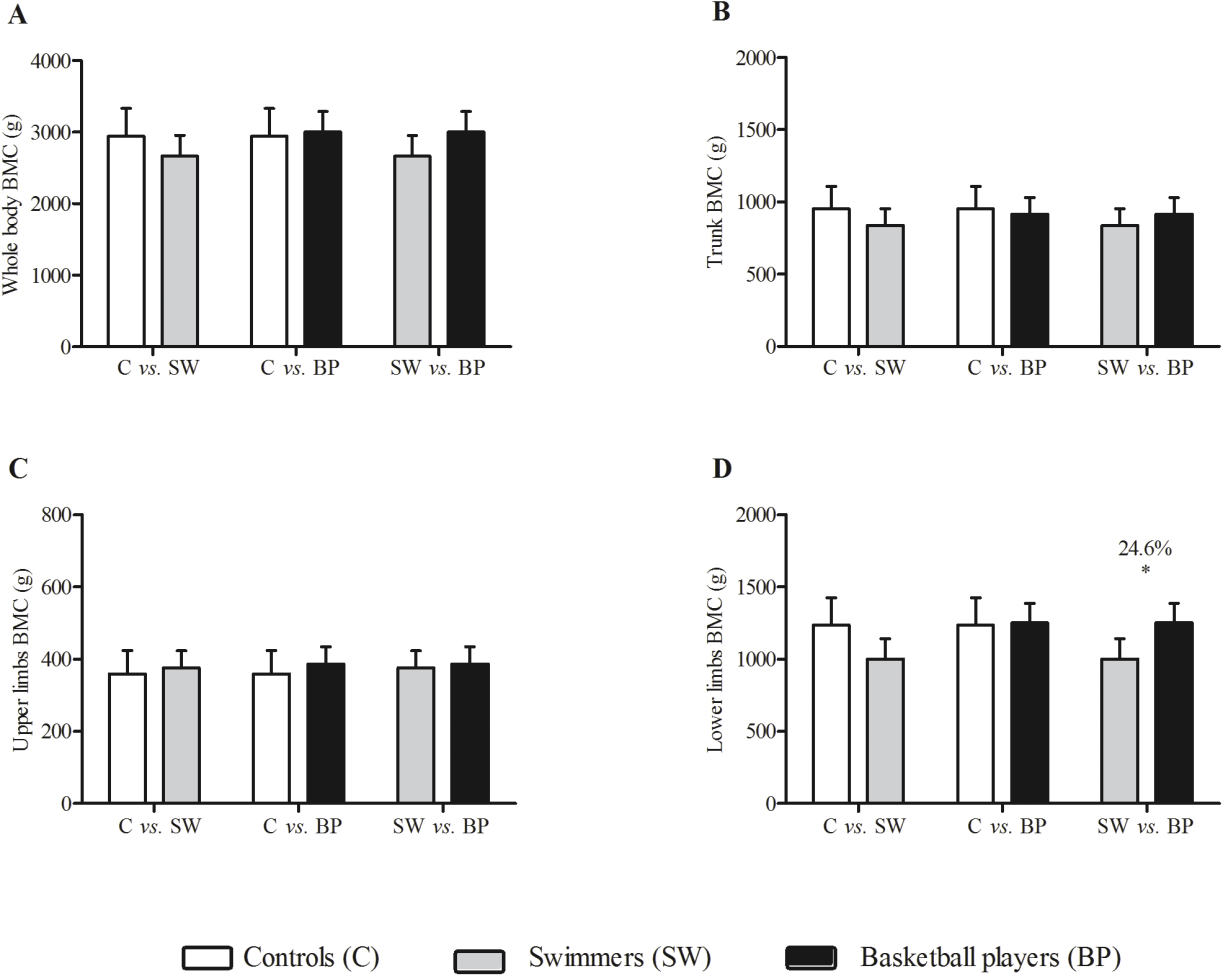
Whole body bone mineral content (BMC), trunk BMC, upper limbs BMC, lower limbs BMC in male controls (white bars), swimmers (grey bars) and basketball players (black bars) adjusted by chorological age, maturity offset, vitamin D intake and weekly training load. * indicates difference between the groups (p<0.05).

**Fig 4:**
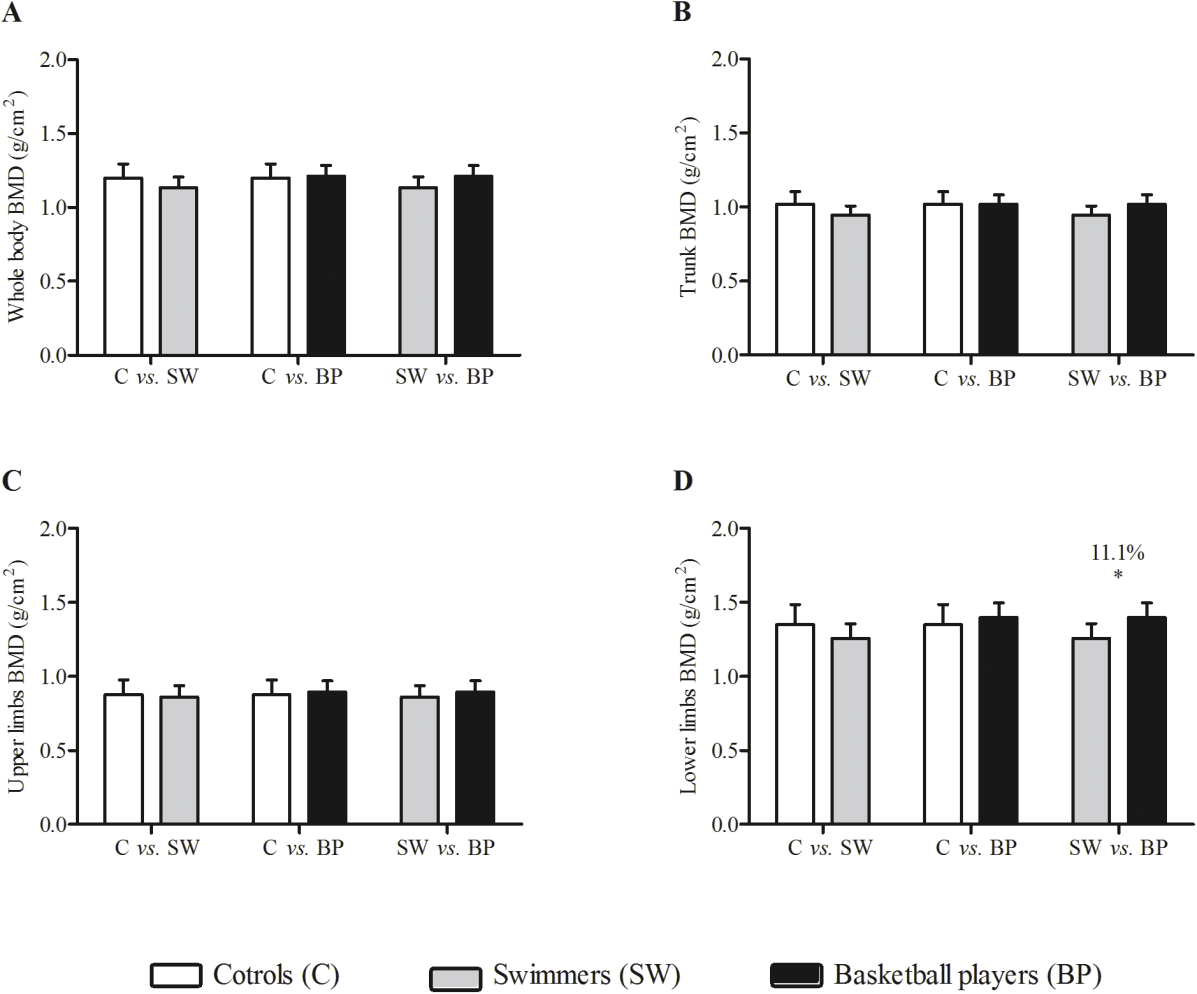
Whole body bone mineral content (BMD), trunk BMD, upper limbs BMD, lower limbs BMD in male controls (white bars), swimmers (grey bars) and basketball players (black bars) adjusted by chorological age, maturity offset, vitamin D intake and weekly training load. * indicates difference between the groups (p<0.05).

Multiple comparisons between groups were presented in Tables 5 and 6 and Figs 2–4. Among blood parameters, only VLDL and triglycerides showed a moderate effect between controls and athletes. The comparisons of controls with other groups were very large for H-S CRP (*d* range: 2.17–2.92; difference between groups: 185.7–290.4%). Regarding bone mineral density, the differences between controls and the other two groups were moderate when compared to swimmers (*d* = 1.10) and very large when compared to basketball players (*d* = 2.19).

**Table 5:** Mean difference between groups on chronological age, maturation, training experience, parameters of training load, anthropometry of the overall body size, indicators of lipid profile plus inflammatory biomarker and outputs of whole body DXA.

Dependent variable	Unit	Mean differences between groups								
		Controls vs. Swimmers			Controls vs. Basketball players			Swimmers vs. Basketball players		
		mean dif. (95% CI)	d	qualitative	mean dif. (95% CI)	d	qualitative	mean dif. (95% CI)	d	qualitative
Total cholesterol	mg/dL	2.34 (-15.71; 20.39)	0.12	(trivial)	15.43 (-1.73; 32.60)	0.68	(small)	13.09 (-5.38; 31.57)	0.58	(small)
HDL-c	mg/dL	-6.63 (-13.09; -0.17)	0.95	(moderate)	-0.96(-7.10; 5.19)	0.14	(small)	5.67 (-0.94; 12.28)	0.64	(small)
LDL-c	mg/dL	0.75 (-14.50; 16.01)	0.07	(trivial)	8.49 (-6.01; 23.01)	0.56	(small)	7.74 (-7.87; 23.36)	0.45	(small)
VLDL-c	mg/dL	8.21 (2.30; 14.12)	1.01	(moderate)	7.89 (2.27; 13.52)	0.98	(moderate)	-0.32 (-6.37; 5.73)	0.10	(trivial)
Triglycerides	mg/dL	41.10 (11.56; 70.63)	1.01	(moderate)	39.44 (11.35; 67.53)	0.98	(moderate)	-1.65 (-31.88; 28.57)	0.10	(trivial)
HS C-reactive protein	mg/L	-2.53 (-3.92; -1.15)	2.92	(very large)	-3.97 (-5.26; -2.67)	2.17	(very large)	-1.43 (-2.71; -0.15)	0.78	(moderate)
DXA-Whole body										
Total tissue	kg	-5.62(-13.85; 2.61)	0.58	(moderate)	-15.07 (-22.90; -7.24)	1.72	(large)	-9.45 (-19.59; 0.68)	0.89	(moderate)
Fat tissue	kg	9.93 (4.23; 15.64)	1.37	(large)	7.61 (2.19; 13.04)	1.04	(moderate)	-2.32 (-10.04; 5.40)	0.44	(small)
Lean soft tissue	kg	-14.88 (-21.02; -8.74)	2.03	(very large)	-21.51 (-27.35; -15.67)	3.12	(very large)	-6.63 (-13.05; -0.21)	0.88	(moderate)
BMC *	g	276 (-493; 1046)	-	—	-60 (-838; 717)	-	—	-336 (-691; 18)	-	—
Bone area *	cm^2^	45 (-721; 813)	-	—	-48 (-823; 726)	-	—	-94 (-448; 259)	-	—
BMD *	g/cm^2^	0.065 (-0.125; 0.255)	-	—	-0.015 (-0.207; 0.177)	-	—	-0.080(-0.167; 0.008)	-	—

Abbreviations: mean dif., mean difference; 95%CI, 95% confidence interval; d, Cohen's d; HDL-c, high-density lipoprotein cholesterol; LDL-c, low-density lipoprotein cholesterol; VLDL-c, very-low-density lipoprotein cholesterol; HS C-reactive protein, high-sensitivity C-reactive protein; DXA, dual-energy X-ray absorptiometry; BMC, bone mineral content; BMD, bone mineral density.

* adjusted by chronological age, maturity offset, score of vitamin D intake and weekly training volume. The cohen’s d was withdrawn because adjusted means did not present standard deviation required for the calculation.

**Table 6:** Mean difference between groups on DXA assessments on trunk, upper limbs and lower limbs.

Dependent variable	Unit									
		Controls vs. Swimming			Controls vs. Basketball			Swimming vs. Basketball		
		mean dif. (95% CI)	d	qualitative	mean dif. (95% CI)	d	qualitative	mean dif. (95% CI)	d	qualitative
***Total tissue***										
Trunk	kg	-3.94 (-7.81; -0.07)	0.82	(moderate)	-6.67 (-10.35; -2.99)	1.71	(large)	-2.72 (-7.37; 1.91)	0.55	(small)
Upper limbs	kg	-2.07 (-3.15; -0.99)	1.60	(large)	-2.67 (-3.70; -1.64)	2.36	(very large)	-0.60 (-1.82; 0.62)	0.43	(small)
Lower limbs	kg	0.40 (-2.92; 3.73)	0.11	(trivial)	-5.30 (-8.46; -2.13)	1.39	(large)	-5.70 (-9.90; -1.51)	1.35	(large)
***Fat tissue***										
Trunk	kg	4.05 (1.16; 6.94)	1.10	(moderate)	3.24 (0.49; 5.99)	0.90	(moderate)	-0.81 (-4.00; 2.37)	0.29	(small)
Upper limbs	kg	0.76 (0.33; 1.20)	1.32	(large)	0.71 (0.30; 1.12)	1.25	(moderate)	-0.06 (-0.55; 0.43)	0.18	(trivial)
Lower limbs	kg	4.76 (2.40; 7.11)	1.62	(large)	3.37 (1.13; 5.61)	1.09	(moderate)	-1.39 (-4.03; 1.25)	0.66	(moderate)
***Lean soft tissue***										
Trunk	kg	-7.73 (-10.52; -4.95)	2.32	(very large)	-9.50 (-12.15; -6.84)	3.09	(very large)	-1.76 (-4.62; 1.10)	0.51	(small)
Upper limbs	kg	-2.66 (-3.64; -1.68)	2.27	(very large)	-3.19 (-4.12; -2.26)	2.93	(very large)	-0.53 (-1.51; 0.46)	0.44	(small)
Lower limbs	kg	-4.18 (-6.52; -1.83)	1.55	(large)	-8.16 (-10.39; -5.93)	3.01	(very large)	-3.98 (-6.49; -1.47)	1.39	(large)
Bone mineral content*										
Trunk	g	114.3 (-194.0; 422.8)	-	—	-36.5 (-275.1; 348.1)	-	—	-77.8 (-220.1; 64.3)	-	—
Upper limbs	g	-15.5 (-141.5; 110.4)	-	—	-27.4 (-154.7; 99.8)	-	—	-11.8 (-69.9; 46.2)	-	—
Lower limbs	g	235.2 (-131.8; 602.4)	-	—	-12.1 (-383.1; 358.8)	-	—	-247.4 (-416.8; -78.1)	-	—
Bone area*										
Trunk	cm^2^	30.3 (-154.8; 215.6)	-	—	15.8 (-171.3; 202.9)	-	—	-14.5 (-99.9; 70.8)	-	—
Upper limbs	cm^2^	-38.9 (-144.8; 66.9)	-	—	-39.9(-146.9; 67.0)	-	—	-0.97 (-49.8; 47.8)	-	—
Lower limbs	cm^2^	103.7 (63.0; 270.6)	-	—	13.8 (-154.7; 182.3)	-	—	-89.9 (-166.9; -13.0)	-	—
Bone mineral density*										
Trunk	g/cm^2^	0.074 (-0.093; 0.242)	-	—	-0.002 (-0.171; 0.168)	-	—	-0.076 (-0.154; 0.001)	-	—
Upper limbs	g/cm^2^	0.014 (-0.187; -0.215)	-	—	-0.020 (-0.223; -0.183)	-	—	-0.034 (-0.126; 0.059)	-	—
Lower limbs	g/cm^2^	-0.093 (-0.174; 0.360)	-	—	-0.048 (-0.318; 0.223)	-	—	-0.141 (-0.264; -0.017)	-	—

Abbreviations: DXA, dual-energy X-ray absorptiometry; mean dif., mean difference; 95%CI, 95% confidence interval; d, Cohen's d.

* adjusted by chronological age, maturity offset, score of vitamin D and weekly training volume. The cohen’s d was withdrawn because adjusted means did not present standard deviation required for the calculation.

The comparisons between swimmers and basketball players presented small to trivial differences for the metabolic parameters, with the exception of HS-CRP (*d* = 0.8; difference between groups: 36.7%). Regarding tissue outputs obtained from DXA scan, differences between sport disciplines were interpreted as moderate for all variables except fat tissue (*d* = 0.4). While considering different sites in the assessment of bone variables, it was possible to determine small differences (*ES-r* = 0.17) between controls and swimmers for bone area at the lower limbs (103.7 cm^2^, 13.0%). In parallel, between swimmers and basketball players, the gradient of the differences was small (*ES-r* range: 0.15–0.23) for bone mineral content (247.4 g, 24.6%), bone area (89.9 cm^2^, 11.3%) and bone mineral density (0.141 g/cm^2^, 11.1%) at the lower limbs, favoring the basketball players.

## Discussion

The present study presents the opportunity to deepen the knowledge of exercise-related effects on bone and blood health in youth, including participants of different typologies of sports. In particular, basketball is considered to rely predominantly on the anaerobic metabolism to perform short all-out jumping and sprint performances [29], whereas swimming involves cyclic sequences primarily supported by the aerobic metabolism [30, 31]. The main findings of the present study were that youth athletes presented a better BMI, blood health with respect to controls. Although swimmers and basketball players showed more lean soft tissue and also an advanced maturity offset, after adjustments for potential confounders (i.e., chronological age, maturity offset, vitamin D and training per week [minutes]) the bone content and density were similar to the observed in control group. Comparing the sport participation groups, differences emerged for the lower limbs, with basketball players presenting higher BMD and BMC compared to swimmers.

Sport-specific stimuli could affect bone parameters. In fact, tennis players presented high bone mass at the distal portion of the radius [32], whereas players showed high bone mass mainly in the femur [12]. The present findings provide novel information of youth basketball players and swimmers. In line with the literature [33], basketball players showed the highest stature values, substantiating the knowledge that this sport favours the selection of tall athletes, but also the highest bone area, BMC, and BMD, especially evident for the lower limbs. In fact, basketball can be considered as a "high-impact" sport, which requires high intensity sprints and abrupt decelerations, vertical jump-landings, lay-ups, and shooting [34] resulting in mechanical loads that stimulate the bone remodeling process and an increased bone health of lower limbs [34, 35] unlike swimming which is performed in hypogravity and does not promote mechanical stimulation during its practice [36]. However, when comparing with the control group, although basketball athletes tended to higher values of bone variables, the results were not significantly different between the groups. These results can be explained by the fact that sport participation tends to influence bone mass gain during the entire adolescence (until 18–20 years) and the mean age of the basketball players was 14.5 ± 0.9 years. Thus, longer follow-up periods may be able to show a deeper understanding of the impact of sport in bone health [37].

In adults a regular practice of physical exercise is associated to improvements in blood lipid profile [38] and reductions of the risks of non-communicable diseases [39], but in children and adolescents the evidence is still unclear because most studies have been accomplished on single interventions, often focused on youth presenting obesity and/or metabolic disorders [40]. In this study, the lack of differences between groups on total cholesterol and LDL indicates that 2-hour^.^week^-1^ school physical education could protect healthy youth, independently of sport practice. Conversely, sport practice can provide additional positive effects on the other blood parameters. In fact, athletes presented better VLDL and triacylglycerol profiles with respect to the control group, probably due to increased requirements of free fatty acids for oxidation and utilization for sustaining training [41–43]. Furthermore, sport-specific demands could differentiate other blood parameters. Actually, the highest concentration of HDL in swimmers seems to substantiate that aerobic exercises could be more effective in increasing HDL [44], whereas the highest HS-CRP in basketball players might indicate that this sport induces a pronounced inflammatory response [45].

Despite these findings support the positive effects of swimming and basketball on blood profiles and bone health of youth athletes, the cross-sectional design of this study allows only speculations and limits the possibility to prove causality. Furthermore, the lack of information on nutrition, hormonal status, other inflammatory and anti-inflammatory markers, also should be considered a potential limitation that need further investigations.

## Conclusions

In summary, sport participation has been linked to beneficial differences in terms of lipids and bone parameters among male adolescents. Basketball seems more beneficial to bone parameters (mainly in lower limbs) than swimming, while metabolic parameters (lipids) seem more likely to be affected by swimming participation than basketball. Thus, considering that competitive sport is deeply linked to active lifestyles and represents an important opportunity to implement physical activity [46–48] and to counteract the secular trends of increasing non-communicable diseases [49] and of youth inactivity in western countries [50], policy makers should enforce sport practice of youth individuals. Physical education and sport programs should include aerobic and anaerobic activities that enhance the possibility to ameliorate blood profiles of youth but it is necessary caution in the choose of the most appropriated sport when targeting improvements in bone health.

## Supporting information

S1 File Full dataset. (XLSX)
